# Targeting PI3K/AKT/mTOR Pathway Exerts Inhibitory Effects on Endothelial Cells from Generalized Lymphatic Anomaly

**DOI:** 10.3390/cells15201860

**Published:** 2026-10-13

**Authors:** Bruno Lorusso, Laura Finato, Antonella Nogara, Giovanni Roti, Anna Montanaro, Gregorio Monica, Alice Andreani, Gallia Graiani, Pellegrino Crafa, Enrico Maria Silini, Letizia Gnetti, Francesco Paolo Pilato, Lorena Bottarelli, Cinzia Azzoni, Giuseppe Cerasoli, Federico Quaini, Costanza Anna Maria Lagrasta

**Affiliations:** 1 Department of Medicine and Surgery, University of Parma, 43126 Parma, Italy; brunolorusso@gmail.com (B.L.); giovanni.roti@unipr.it (G.R.); anna.montanaro@unipr.it (A.M.); pellegrino.crafa@unipr.it (P.C.); enricomaria.silini@unipr.it (E.M.S.); lorena.bottarelli@unipr.it (L.B.); cinzia.azzoni@unipr.it (C.A.); federico.quaini@unipr.it (F.Q.); costanzaannamaria.lagrasta@unipr.it (C.A.M.L.); 2 Translational Hematology and Chemogenomics (THEC), University of Parma, 43126 Parma, Italy; 3 Hematology and BMT Unit, University Hospital of Parma, 43126 Parma, Italy; 4 Dental School, University of Parma Medical School, 43126 Parma, Italy; gallia.graiani@unipr.it; 5 Pathology Unit, University Hospital of Parma, 43126 Parma, Italy; letyg70@hotmail.com (L.G.); pillogro@gmail.com (F.P.P.); 6 Pediatric Surgery, Ospedale dei Bambini of Parma, University Hospital of Parma, 43126 Parma, Italy; gcerasoli@yahoo.it

**Keywords:** generalized lymphatic anomaly, lymphatic endothelial cells, PI3Kca mutation, immunohistochemistry, alpelisib, rapamycin, dactolisib, buparlisib, migration, angiogenesis

## Abstract

Generalized lymphatic anomaly (GLA) is a congenital, non-neoplastic vascular anomaly characterized by diffuse or multifocal involvement and infiltration of multiple organs. Most GLA cases harbor “hotspot” somatic missense single nucleotide mutations in the *PIK3CA* gene, whereas a minority involve mutations in other genes. Recently, rapamycin and its homologs have shown encouraging results in the treatment of alterations affecting the PI3K/AKT/mTOR pathway. Therefore, the present work aimed to expand the immunophenotypic characterization of the lymphatic lesion at the tissue level and to evaluate the in vitro viability, migration, and angiogenic capacity of endothelial cells freshly isolated from a *PIK3CA*-mutated GLA sample, following treatment with alpelisib, rapamycin, dactolisib, and buparlisib. We detected the p.Gln546Lys amino acid substitution in the PI3Kca helical domain in GLA-derived endothelial cells. Then, by immunohistochemical analysis, we documented altered expression of mTOR and its well-characterized downstream substrates, which regulate growth, proliferation, migration, angiogenesis, and survival of ECs. Finally, pharmacological treatment reduced GLA-EC functional properties and down-modulated the hyperactivated PI3K downstream substrates. Overall, our results suggest that routine immunohistochemistry, integrated with phospho-protein analysis and next-generation sequencing, might improve histopathological classification of vascular anomalies and facilitate the development of personalized targeted therapies, particularly in recurrent or surgically challenging cases.

## 1. Introduction

Post-zygotic mosaic gain-of-function mutations in the *PIK3CA* gene designate and encompass the so-called *PIK3CA*-related overgrowth spectrum (PROS), an illness group featuring from single benign anomalies to multisystem disorders [1,2]. PROS diseases include single and isolated lymphatic malformation (LM), generalized lymphatic anomaly (GLA), venous malformation (VM), Klippel–Trenaunay syndrome (KTS), congenital lipomatous overgrowth with vascular, epidermal, and skeletal anomalies (CLOVES) syndrome, fibro-adipose vascular anomaly (FAVA), and megalencephaly–capillary malformation (MCAP) syndrome. *PIK3CA* gene encodes the 110 kD catalytic α-subunit (p110α) of phosphatidylinositol 3-kinase (PI3K), an upstream key element that converts phosphatidylinositol (3,4)-bisphosphate (PtdIns(3,4)P2) to phosphatidylinositol (3,4,5)-triphosphate (PtdIns(3,4,5)P3). These proteins lead to the activation of PDK1, which phosphorylates AKT at Thr308. Then, AKT activates the kinase mammalian target of rapamycin (mTOR). The PI3K/AKT/mTOR pathway regulates multiple cellular functions including proliferation, growth, survival, angiogenesis, and cell motility [3,4,5]. In the last decade, “hot spot” somatic missense single nucleotide mutations of the *PIK3CA* gene have been associated with vascular lesions. The most common are high-activating mutations coding for either the helical domain or the kinase domain of the PI3K protein, with a range of mosaicism between 1.1% and 23.0%. Specifically, in mutations involving the helical domain, glutamic acid (Glu), and glutamine (Gln) are replaced by lysine (Lys) at amino acid position 542, 545, and 546, respectively (p.Glu542Lys, p.Glu545Lys, and p.Gln546Lys), while in mutations of the kinase domain, histidine (His) is replaced by arginine (Arg) or leucine (Leu) at amino acid position 1047 (p.His1047Arg and p.His1047Leu) [6,7,8,9]. Other rare lymphatic anomalies may involve mutations in the RAS/MAPK pathway, another intracellular signaling pathway able to generate overgrowth disorders [10,11,12]. In addition, mutations of the *PIK3CA* gene are widely reported in several human cancers, although they mainly co-occur with mutations in other genes [13,14,15]. Moreover, in recent years, rapamycin and its homologs, and other target inhibitors of individual steps of the PI3K/AKT/mTOR pathway, have been employed as anti-cancer agents. Likewise, by this approach, vascular anomalies have been treated with encouraging outcomes. However, therapeutic interventions, including surgical resection, sclerotherapy with bleomycin, laser therapy, and radiofrequency ablation, are needed to prevent or treat complications such as functional impairment, disfigurement, scarring, and local pain [16,17,18,19,20,21].

In particular, the generalized lymphatic anomaly (GLA) is characterized by abundant dilated, tortuous, dysmorphic lymphatic channels lined with flattened endothelial cells (ECs). On gross examination, GLA consists of abnormal lymphatic vessels with both macrocystic and microcystic components, or their combination, filled with lymph that can infiltrate many organs, such as the skin, abdominal wall, soft tissues, and bones [9,21,22].

The aims of the present study were: *(1)* to improve the immunophenotypic characterization of GLA at the tissue level and *(2)* to test at molecular and functional levels the in vitro pharmacological interference of the PI3K/AKT/mTOR pathway on GLA-derived endothelial cells (GLA-ECs). Thus, we isolated and cultured lymphatic endothelial cells from a GLA sample obtained from a pediatric patient. By next-generation sequencing (NGS) performed on GLA-ECs, we reported a *PIK3CA* mutation in exon 10: c.1636C>A, resulting in a p.Gln546Lys substitution in the amino acid sequence of the PI3Kca protein. After in vitro pharmacological treatment of GLA-ECs, viability by MTT, angiogenesis on Matrigel^®^, and migration by wound-healing assay were evaluated. Finally, we investigated the PI3K/AKT/mTOR downstream signaling pathway by Western blot.

## 2. Materials and Methods

### 2.1. Sample Collection

Generalized lymphatic anomaly (GLA) and human foreskin samples were obtained from the Unit of Pediatric Surgery—Ospedale dei Bambini of Parma. Biological samples were collected and transferred to the Cell Culture Laboratory, Pathology Unit of the University Hospital of Parma, under sterile conditions within 30 min of resection. In particular, the GLA specimen was sampled by medical staff ensuring the priority of their use for diagnostic purposes. Human umbilical cords from pregnancies to term were obtained from the Unit of Obstetrics and Gynecology of the University Hospital of Parma. Patients were enrolled after parental informed consent for the use of biological samples for research purposes. The study was conducted in accordance with the Declaration of Helsinki and was approved by the Ethics Committee of Area Vasta Emilia Nord (CE AVEN; protocol code 129/2018/OSS/AOUPR).

### 2.2. Histological and Immunohistochemical Staining

Hematoxylin and eosin (H&E) and immunohistochemical (IHC) staining were performed on 3.5 µm sections of formalin-fixed paraffin-embedded (FFPE) tissues from GLA and human foreskin samples and then deparaffinized and rehydrated through xylene and decreasing ethanol solutions. The primary antibodies employed for immunoperoxidase staining are shown in Table 1. All sections were revealed by UltraView Universal DAB Detection Kit (Ventana, Export, PA, USA) or IHC Detection Kit-Micropolymer (Abcam, Cambridge, UK; ab236466) according to the manufacturer’s recommendations [23]. Hematoxylin was used as the counterstain.

For immunofluorescence staining, 3.5 µm sections from FFPE GLA and human foreskin tissues were incubated after rehydration with goat serum (Merck, Rahway, NJ, USA; G9023) for 30 min at room temperature (RT) to block aspecific binding followed by mouse anti-human podoplanin/D2-40 (Cell Marque, Rocklin, CA, USA; 322M-18; ready-to-use; 30 min at 37 °C), rabbit anti-human phospho-mTOR^Ser2448^ (Cell Signaling Technology, Danvers, MA, USA; #5536; 1:50; o/n at 4 °C), or rabbit anti-human phospho-rpS6^Ser235/236^ (Cell Signaling Technology; #4858; 1:150; o/n at 4 °C). Sections were then incubated with goat anti-rabbit IgG fluorescein isothiocyanate-conjugated (FITC) (Jackson ImmunoResearch Laboratories, Inc., West Grove, PA, USA; 111-545-003; 1:400; 60 min; 37 °C) or goat anti-mouse IgG tetramethylrhodamine isothiocyanate (TRITC) (Merck; T6528; 1:50; 60 min; 37 °C)-specific secondary antibodies. Next, coverslips were mounted with Vectashield containing 4′,6-Diamidino-2-phenylindole (DAPI) (Vector Laboratories, Newark, CA, USA; H-1200). Immunohistochemistry also involved the detection of vascular endothelial growth factor receptor-2 and -3 (VEGFR-2, VEGFR-3), lymphatic vessel endothelial hyaluronan receptor 1 (LYVE-1), prospero-related homeobox gene transcription factor (PROX1), phospho-mTOR^Ser2448^, phospho-AKT^Ser473^, phospho-eukaryotic translation initiation factor 4E-binding protein 1 (also known as p-4E-BP1^Ser65^), phospho-p70 S6 kinase (p-p70S6K^Thr389^), and phospho-ribosomal protein S6 (p-rpS6^Ser235/236^). All staining procedures were repeated at least two times, yielding similar results.

The evaluation of immunostaining was performed independently by four pathologists (E.M.S., F.P.P., L.G., and P.C.) using a light/fluorescence microscope (Olympus BX60, Tokyo, Japan) connected with a digital camera (QICAM, QImaging, Surrey, BC, Canada).

### 2.3. Isolation of Endothelial Cell Lines

Following our previously described protocols [24,25], fragments of the GLA and foreskin samples were finely minced under sterile conditions using surgical scissors and subjected to enzymatic digestion for 60 min at 37 °C with 1 mg/mL collagenase/dispase solution (Roche Diagnostics, Basel, Switzerland; 11097113001). The resulting digestion product was filtered using a 100 µm cell strainer (BD Biosciences, San Jose, CA, USA; 352360) to remove aggregates. The harvested cells were washed, seeded on a 6-well plate (Corning, Corning, NY, USA; 3516) pre-treated with collagen solution type I (Merck; C8919), and cultured in complete medium composed of Endothelial Cell Growth Medium (ready to use; Cell Application, Inc., San Diego, CA, USA; 211-500) plus 100 U/mL penicillin and 100 µg/mL streptomycin (Merck; P4333). After 5-7 days, heterogeneous adherent cell populations were recovered with trypsin/EDTA (Merck; T3924) and sorted with anti-human CD31 immunolabeled magnetic microbeads (CD31 MicroBeads Kit; Miltenyi Biotec, Bergisch Gladbach, Germany; 130-091-935), according to the manufacturer’s recommendations. Harvested CD31^pos^ cells, defined as generalized lymphatic anomaly-derived endothelial cells (GLA-ECs) and human normal dermal-derived endothelial cells from foreskin (HD-ECs), were cultured in complete medium.

Human umbilical vein endothelial cells (HUVECs) were recovered by enzymatic digestion. In brief, one end of the vein was cannulated with a blunt-tip steel needle to wash the lumen with Dulbecco’s phosphate-buffered saline (DPBS; Thermo Fischer Scientific, Waltham, MA, USA; 14190144). Subsequently, 1 mg/mL pre-warmed (37 °C) collagenase/dispase solution was injected and allowed to incubate for 30 min at RT. The ECs were recovered, flushing the vein lumen with 3 × 10 mL DPBS, washed, centrifuged, and seeded in Endothelial Cell Growth Medium plus 100 U/mL penicillin and 100 µg/mL streptomycin. Human Dermal Lymphatic Endothelial Cells (HD-LECs; PromoCell, Heidelberg, Germany; C-12217), commercially available primary cells, were cultured in Endothelial Cell Growth Medium v2 (Cell Applications, Inc.; 213-500; ready-to-use).

GLA-ECs, HD-ECs, HUVECs, and HD-LECs were incubated at 37 °C in a humidified atmosphere containing 5% CO_2_ and used at passages 2 to 7 for all the experiments. All endothelial cell lines were tested for mycoplasma contamination at the beginning and during all experiments by MycoStrip^TM^ (Invivogen, San Diego, CA, USA; rep-mys-100), according to the manufacturer’s protocol.

### 2.4. Immunocytochemistry/Immunofluorescence

GLA-ECs, cultured on chamber slides (BD Biosciences; 354114) coated with collagen solution type I, were fixed (4% paraformaldehyde (PFA) in DPBS, pH = 7.4 for 15 min at RT) and incubated with the following primary unconjugated antibodies: mouse anti-human CD31 (Agilent, Santa Clara, CA, USA; M0823; 1:50; 30 min; 37 °C) and mouse anti-human D2-40 (Biocare Medical, Pacheco, CA, USA; CM266A; 1:50; 30 min; RT). The immunoreaction was revealed by IHC Detection Kit-Micropolymer according to the manufacturer’s recommendations. Cytological preparations were then counterstained with hematoxylin, closed with coverslips, and analyzed by an optical microscope (Olympus BX60) connected to a QICAM camera. For PROX1 staining, the GLA-EC monolayer was fixed and permeabilized for 15 min with 4% PFA plus 0.3% Triton-X100 (Merck; T8787) solution. Then, rabbit anti-human PROX1 primary antibody was used (Origene, Rockville, MD, USA; DP3516P; 1:300; 4 °C; o/n), followed by goat anti-rabbit IgG FITC-conjugated secondary antibody (Jackson ImmunoResearch Laboratories, Inc.; 111-545-003; 1:400; 60 min; 37 °C). Finally, to evaluate the purity of the isolated HD-ECs and HUVECs, confluent monolayers were stained with mouse anti-human CD31 primary unconjugated antibody (Agilent; M0823; 1:50; 30 min; 37 °C) followed by goat anti-mouse IgG FITC-conjugated secondary antibody (Jackson ImmunoResearch Laboratories, Inc.; 115-545-003; 1:400; 60 min; 37 °C). Slides were mounted with Vectashield containing DAPI and analyzed using a fluorescence microscope (Olympus BX60) connected to a QICAM camera. Negative controls were represented by exposing cell preparations to the same immunostaining procedure but excluding primary antibodies.

### 2.5. Flow Cytometric Analysis

To further characterize the endothelial cell phenotype, 5 × 10^5^ GLA-ECs and HD-ECs were incubated with 80 µL of ready-to-use mouse anti-human podoplanin/D2-40 antibody (Cell Marque; 322M-18), followed by incubation with goat anti-mouse IgG fluorescein isothiocyanate (FITC)-conjugated secondary antibody (Jackson ImmunoResearch Laboratories, Inc.; 115-545-003; 1:200). Both incubations were performed for 30 min at 37 °C in the dark.

To assess cell proliferation following pharmacological treatment, at least 2 × 10^5^ GLA-ECs, either untreated or treated with 1 µM BYL, 25 nM RAPA, 25 nM BEZ, or 1 µM BKM for 24 or 48 h, were harvested and fixed/permeabilized using the BD Cytofix/Cytoperm™ Fixation/Permeabilization Kit (BD Biosciences; 554714), according to the manufacturer’s instructions. Cells were subsequently incubated with 2 µL of anti-human ki-67-FITC REAfinity™ antibody (Miltenyi Biotec, Bergisch Gladbach, Germany; 130-117-691) for 30 min at 4 °C in the dark.

Negative controls consisted of cells subjected to the same staining procedures with omission of the primary and/or secondary antibody, as appropriate. For each experimental condition, at least 2 × 10^4^ events were acquired using an Attune™ NxT Acoustic Focusing Cytometer (Invitrogen, Thermo Fisher Scientific, Waltham, MA, USA). Cell doublets were excluded using an FSC-A versus FSC-H gate, followed by selection of the main cell population based on FSC-A versus SSC-A characteristics. D2-40 and ki-67-positive cells were then quantified within the gated singlet population. Data were analyzed using FlowJo v10.8 software (BD Biosciences).

### 2.6. Next-Generation Sequencing

DNA was extracted from 1 × 10^6^ cultured GLA-ECs (Qiagen, Germantown, MD, USA) and analyzed by next-generation sequencing (NGS) using Myriapod NGS Cancer Panel Plus (Diatech Pharmacogenetics s.r.l., Jesi, Italy) on the MiSeq^®^ system (Illumina, San Diego, CA, USA) (Table S1). The sequence alignment in hg19 and the variant calling were performed using Myriapod^®^ NGS Data Analysis Software v.5.0.3 (Diatech Pharmacogenetics s.r.l., Jesi, Italy) with a software detection limit (LoD) of 95%.

### 2.7. Drug Reagents

The pharmacological inhibitors used in this study are summarized in Table 2. All compounds were purchased from Selleckchem (Houston, TX, USA) and were reconstituted and stored according to the manufacturer’s recommendations until use.

### 2.8. MTT Assay

GLA-ECs were seeded at 4 × 10^3^ cells per well in collagen type I-coated 96-well flat-bottom plates (Corning; C3595) in 100 µL of complete medium. After adhesion, ECs were exposed to increasing BYL (0.1, 0.25, 0.5, 1, and 2 µM), RAPA (1, 2.5, 5, 25, and 50 nM), BEZ (5, 10, 25, 50, and 100 nM), or BKM (0.1, 0.25, 0.5, 1, and 2 µM) concentrations for 24 and 48 h. At each time point, 10 µL of 3-(4,5-dimethylthiazol-2-yl)- 2,5-diphenyltetrazolium bromide (MTT) at 5 mg/mL (VWR Life Science, Radnor, PA, USA; 0793-1G) was added to each well. Plates were further incubated at 37 °C for 3 h. At the end of the incubation, the formazan was dissolved using 100 µL of dimethyl sulfoxide (DMSO; Merck, D2650) per well. The optical density was measured with a microplate reader (Victor^TM^X4; Perkin Elmer, Waltham, MA, USA). All experiments were carried out three times, and five replicates for each condition were made.

### 2.9. Apoptosis Assays

For apoptosis analysis, 5 × 10^5^ GLA-ECs were cultured in complete medium either in the absence (CTRL) or presence of 1 µM BYL, 25 nM RAPA, 25 nM BEZ, or 1 µM BKM for 48 h. Cells were then harvested and stained with Annexin V-FITC and propidium iodide (PI) using the eBioscience™ Annexin V Apoptosis Detection Kit (Invitrogen, Thermo Fisher Scientific; BMS500FI-100), according to the manufacturer’s instructions. Apoptosis was assessed using an Attune™ NxT Acoustic Focusing Cytometer. Cell doublets were excluded using an FSC-A versus FSC-H gate, followed by selection of the main cell population based on FSC-A versus SSC-A characteristics. Annexin V-FITC and PI fluorescence was subsequently evaluated within the gated singlet population. Annexin V^−^/PI^−^ cells were considered viable, Annexin V^+^/PI^−^ cells early apoptotic, Annexin V^+^/PI^+^ cells late apoptotic, and Annexin V^−^/PI^+^ cells necrotic. Total apoptosis was calculated as the sum of early apoptotic (Annexin V^+^/PI^−^) and late apoptotic (Annexin V^+^/PI^+^) cell populations. A minimum of 20,000 events was acquired for each experimental condition, and data were analyzed using FlowJo v10.8 software (BD Biosciences).

### 2.10. Wound-Healing Assay

GLA-EC monolayers were cultured in 12-well culture plates (Greiner, Kremsmünster, Austria; 665180), coated with collagen type I solution. Next, GLA-ECs were scraped with a 1000 µL sterile plastic pipette tip and washed, and the culture medium was replaced with complete medium (control group) or with 1 µM BYL, 25 nM RAPA, 25 nM BEZ, or 1 µM BKM for 24 h. Concurrently, GLA-ECs, HD-ECs, and HD-LECs monolayers were cultured in 12-well culture plates in the same conditions, and after the scratch, medium was replaced either with complete medium or low-glucose Dulbecco’s Modified Eagle’s Medium (DMEM; Merck; D6046; with 5.5 mM glucose) plus 0.5% FBS, 1x non-essential amino acid (Gibco, Waltham, MA, USA; 11140-035), 100 U/mL penicillin, and 100 µg/mL streptomycin (starvation medium). Images of the wound area were acquired at 0 and 24 h using an inverted microscope (Olympus CK40) connected to a digital camera (Olympus DP21) and analyzed using ImageProPlus 6.0 software. For each individual scratch, wound closure was normalized to its corresponding initial wound area at 0h. The percentage of healed area was calculated using the formula $\%\;\mathrm{healed}\;\mathrm{area}=\left(\frac{{\mathrm{area}}_{t0}-{\mathrm{area}}_{t24}}{{\mathrm{area}}_{t0}}\right)\times 100$. Experiments were performed three independent times, with four technical replicates for each experimental condition.

### 2.11. In Vitro Lymphangiogenic Assay

Briefly, 100 μL of Matrigel^®^ (Corning; 356231) was loaded at the bottom of each well of the 96-well plates and left for 30 min at 37 °C. GLA-ECs (2 × 10^4^ cells/100 μL in each well) were gently layered on top of Matrigel^®^ in complete medium (control group) or complete medium plus 1 µM BYL, 25 nM RAPA, 25 nM BEZ, or 1 µM BKM. After 24 h, images were captured using an inverted microscope (Olympus CK40) connected to a digital camera (Olympus DP21). All experiments were performed three times, and each experimental condition was repeated three times. In addition, to assess the altered mutation-dependent angiogenic capacity, GLA-ECs, HD-ECs, HUVECs, and HD-LECs monolayers, starved for 18 h, were harvested with trypsin, suspended, counted, and gently layered on top of Matrigel^®^ (2 × 10^4^ cells/100 μL in each well) in complete medium alone or starvation medium. After 6 h, to assess tube-forming ability, images were captured using an inverted microscope (Olympus CK40) connected to a digital camera (Olympus DP21). Capillary-like network formation was quantitatively assessed by counting the number of branching nodes, with each branching node defined as a point of intersection of three or more tubular segments. Branching nodes were counted in each analyzed image using Image-Pro Plus 6.0 software. All experiments were performed twice, and each condition was repeated three times.

### 2.12. Western Blot Assay

GLA-ECs and HD-LECs were seeded in collagen type I-coated 75 cm^2^ flasks (Greiner; C7231) and cultured in their respective complete media. Upon reaching 80% confluence, cell monolayers were washed and incubated in starvation medium for 4 h to minimize interference from exogenous growth factors. Both GLA-ECs and HD-LECs were then either left untreated (CTRL) or treated with 1 µM BYL, 25 nM RAPA, 25 nM BEZ, or 1 µM BKM for 90 min. Procedures for protein extraction, solubilization, and protein analysis by 1-D PAGE are described elsewhere [26]. Briefly, membranes were incubated with primary antibodies: 1:1000 rabbit anti-total ribosomal protein S6 (ABclonal, Woburn, MA, USA; A11874); 1:2000 rabbit anti-phospho-ribosomal protein S6^Ser235/236^ (Cell Signaling Technology; #4858); 1:1000 rabbit anti-total 4E-BP1 (ABclonal; A23500); 1:1000 rabbit anti-phospho-4E-BP1^Ser65^ (Cell Signaling Technology; #9456); 1:1000 rabbit anti-total AKT (Cell Signaling Technology; #4685); 1:750 rabbit anti-phospho-AKT^Ser473^ (Cell Signaling Technology; #3787); and 1:1000 mouse anti-HSP90 (Santa Cruz Biotechnology, Dallas, TX, USA; sc-69703). IRDye 680LT goat anti-mouse IgG (#925-68020) and IRDye 800CW goat anti-rabbit IgG (#925-32211) from LI-COR Biotechnology (Lincoln, NE, USA) were used as secondary species-specific antibodies at a dilution factor of 1:5000. Signals were detected using the ChemiDoc^TM^ MP Imaging System (Bio-Rad Laboratories, Hercules, CA, USA) and quantified with ImageJ v.1.54p software (https://imagej.net/ij/; accessed on 5 October 2026).

### 2.13. Statistical Analysis

Quantitative data are reported as mean ± standard error (SE) and analyzed by one-way analysis of variance (ANOVA) followed by Holm–Sidak’s *post hoc* test. *p* < 0.05 was considered significant.

## 3. Results

### 3.1. Histological and Immunohistochemical Characterization of GLA Tissue

**Figure 1:**
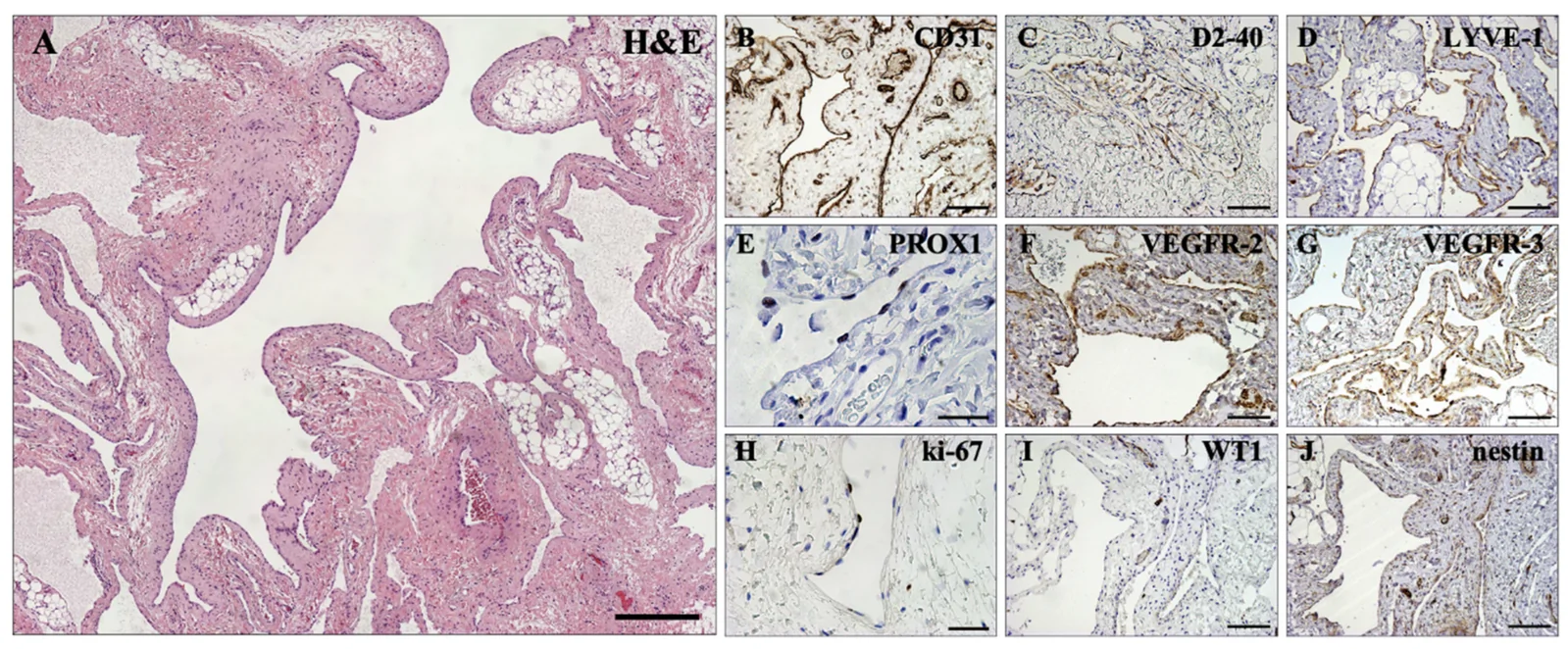
Histological and immunohistochemical analysis of tissue samples from human generalized lymphatic anomaly (GLA). A representative image of an H&E-stained section (**A**) from a GLA sample shows several dilated, convoluted, and poorly organized vessels. Immunoperoxidase-stained sections document the expression of the pan-endothelial marker CD31 (**B**), of lympho-specific antigens D2-40 (**C**) and LYVE-1 (**D**), and of nuclear PROX1 (**E**), as well as of vascular endothelial growth factor receptor-2 and -3 (VEGFR-2/-3; (**F**) and (**G**), respectively). The presence of proliferating endothelial cells is illustrated by the nuclear expression of ki-67 (**H**). To discriminate vascular malformations from vascular neoplasms, serial sections of the same case were immunohistochemically stained for Wilms’ tumor 1 (WT1; (**I**)) and nestin (**J**). Scale bars: (**A**) = 300 μm; (**B**–**D**,**F**,**G**,**I**,**J)** = 100 μm; (**E**) = 30 μm; (**H**) = 50 μm.

The clinical diagnosis of GLA was confirmed by the histological and immunohistochemical analysis. At the tissue level, H&E staining showed numerous dilated, tortuous, dysmorphic lymphatic channels lined with flattened endothelial cells and lumens often filled with lymph. Endothelial cell membranes of the GLA tissue were strongly positive for the pan-endothelial marker CD31 as well as for lymphatic-associated membrane (podoplanin/D2-40 and LYVE-1) and nuclear (PROX1) markers. Moreover, lymphatic lumens were labeled by VEGFR-2 and -3. In addition, vascular profiles were Wilms’ tumor 1 protein (WT1) negative and nestin positive, excluding the neoplastic nature of the vascular lesion, in accordance with the International Society for the Study of Vascular Anomalies and the literature [27,28,29,30]. Finally, increased endothelial cell proliferation in GLA tissue was documented by nuclear ki-67 staining (Figure 1).

### 3.2. Evaluation of PI3K/AKT/mTOR Pathway Activation in GLA Tissue

**Figure 2:**
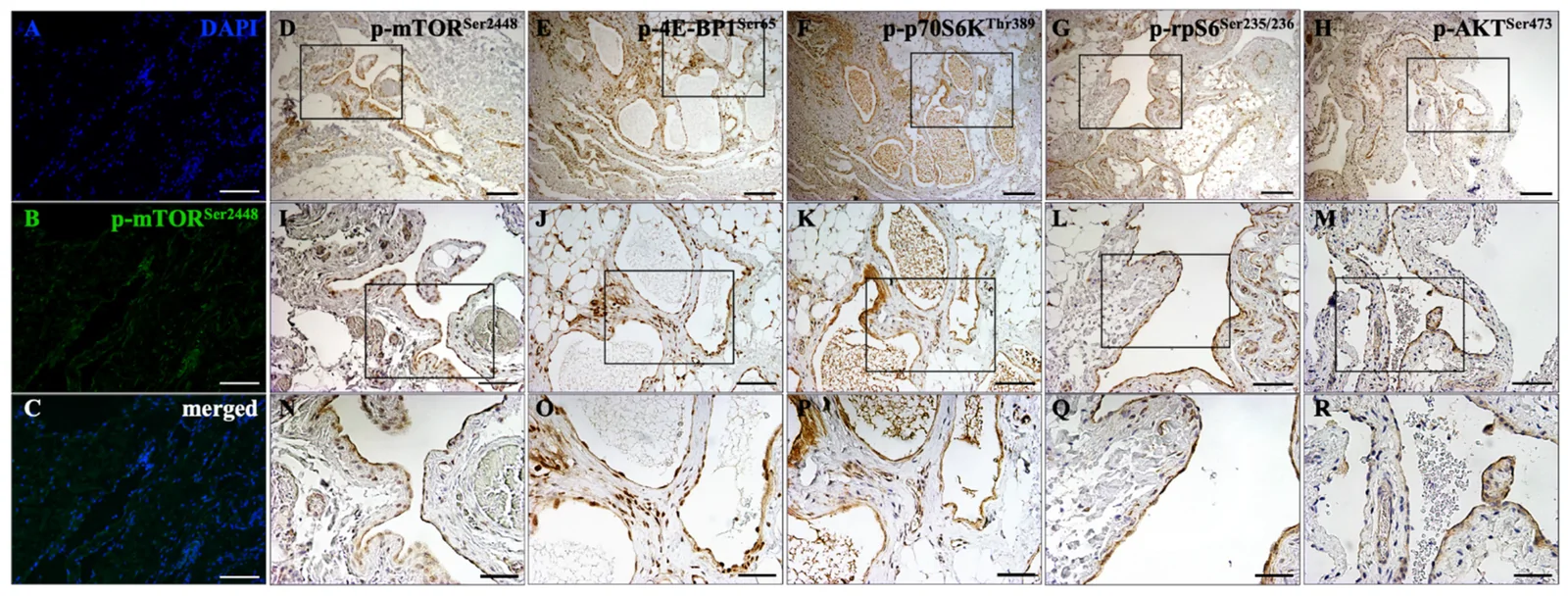
Evaluation of PI3K/AKT/mTOR phosphorylation status on human GLA tissue sample. Representative images of the immunofluorescence and immunohistochemical analysis of tissue samples from human generalized lymphatic anomaly (GLA). Hyperactivated p-mTOR at Ser2448 was documented by both immunofluorescence (**A**–**C**) and immunoperoxidase (**D**,**I**,**N**) using two distinct commercially available antibodies. p-mTOR^Ser2448^-positive lymphatic vessels are shown in green (**B**), with nuclei counterstained with DAPI (blue; (**A**)). p-mTOR^Ser2448^ expression is displayed in brown (**D**,**I**,**N**). Immunoperoxidase staining of p-4E-BP1^Ser65^ (**E**,**J**,**O**), p-p70S6K^Thr389^ (**F**,**K**,**P**), p-rp-S6^Ser235/236^ (**G**,**L**,**Q**), and p-AKT^Ser473^ (**H**,**M**,**R**), well-established downstream effectors of the mTOR axis, further confirmed pathway hyperactivation in GLA tissue. The tissue areas outlined by black rectangles in the low-magnification images (**D**–**H**) are shown at higher magnification in the corresponding panels (**I**–**M**); the areas outlined by black rectangles in (**I**–**M**) are further magnified in the corresponding panels (**N**–**R**). Scale bars: (**D**–**H**) = 200 μm; (**A**–**C**,**I**–**M**) = 100 μm; (**N**–**R**) = 50 μm.

We next examined the phosphorylation status of mTOR at Ser2448, 4E-BP1 at Ser65, p70S6K at Thr389, ribosomal protein S6 at Ser235/236, and AKT at Ser473 in FFPE sections from GLA and human foreskin tissue samples. Positive p-mTOR^Ser2448^ staining was detected in endothelial cells lining GLA vessels, as shown in Figure 2. Conversely, the endothelium of normal human foreskin did not express p-mTOR^Ser2448^ (Figure S1). Positive immunostaining was also observed for p-4E-BP1^Ser65^, a well-characterized direct mTOR target involved in cap-dependent translation initiation. Likewise, p-p70S6K^Thr389^, another key mTOR substrate, and its major downstream target p-rpS6^Ser235/236^ showed positive staining in the endothelial lining of GLA vascular structures. Similar positive staining was observed for p-AKT^Ser473^ (Figure 2). Virtually all vascular profiles within the lymphatic lesion showed positivity for the evaluated phosphorylated signaling proteins. To further confirm that pathway activation occurred in lymphatic endothelial cells, double immunofluorescence staining was performed. Co-localization of D2-40 with phospho-mTOR^Ser2448^ and of D2-40 with phospho-rpS6^Ser235/236^ was observed in GLA vascular structures (Figure S2), confirming activation of the PI3K/AKT/mTOR pathway within the lymphatic endothelial compartment. Consistently, human foreskin control tissues were negative for the evaluated phosphorylated downstream components of the PI3K/AKT/mTOR pathway (Figure S3).

### 3.3. Isolation and Characterization of Endothelial Cell Lines from the GLA Tissue Sample

**Figure 3:**
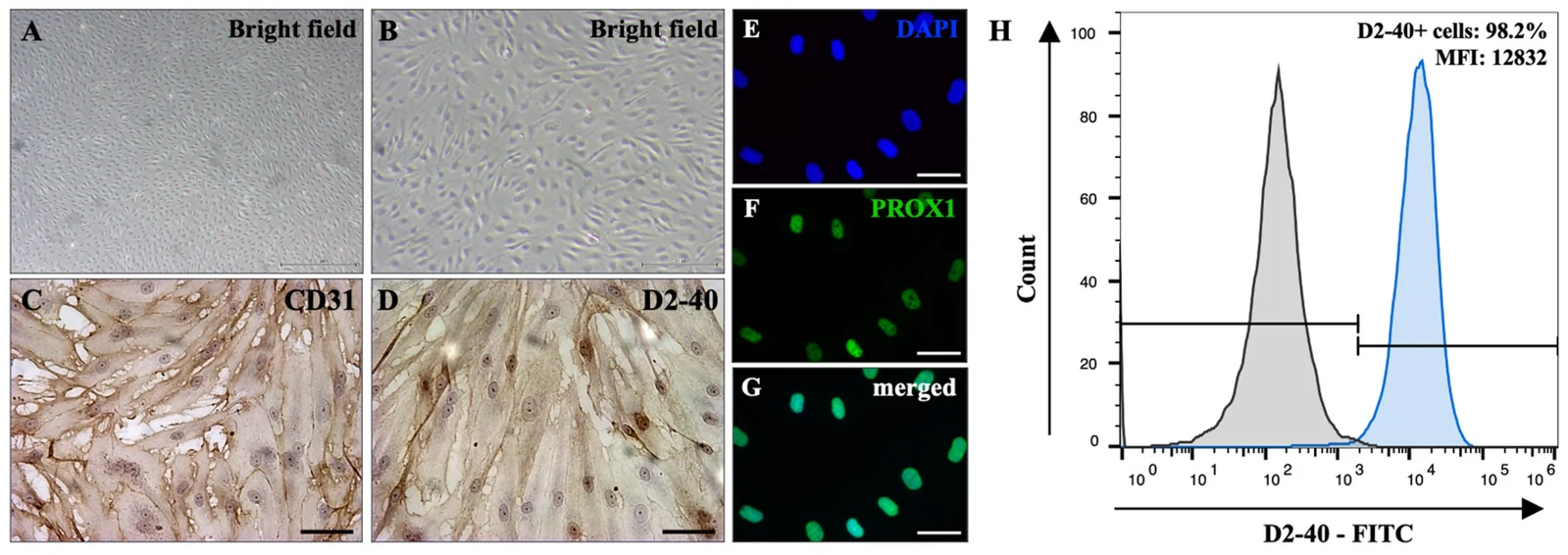
Morphologic and immunophenotypic characterization of generalized lymphatic anomaly-derived endothelial cells (GLA-ECs). A confluent and homogeneous monolayer with cobblestone-like morphology, suggestive of an endothelial phenotype after immunomagnetic sorting, is illustrated at minor and greater magnification, respectively (**A**,**B**). The surface expression of the pan-endothelial marker CD31 (**C**) and lymphatic-associated antigen podoplanin/D2-40 (**D**) is shown in brown by immunoperoxidase detection. Nuclei (blue) are counterstained by hematoxylin. The specific lymphatic lineage commitment of GLA-ECs is documented by the nuclear expression of PROX1 transcription factor (green; (**F**)). Nuclei are counterstained by DAPI (blue; (**E**)). (**G**) Merged image of (**E**,**F**). Scale bars: (**A**) = 500 µm; (**B**) = 200 µm; (**C**,**D**) = 50 µm; (**E**–**G**) = 30 µm. Flow cytometric analysis (**H**) further confirmed D2-40 expression in GLA-ECs, with 98.2% of cells showing D2-40 positivity and a median fluorescence intensity (MFI) of 12832. The unstained control and D2-40-stained population are shown in gray and blue, respectively.

GLA-ECs harvested following enzymatic digestion and immunomagnetic sorting were cultured, expanded, and initially identified as ECs according to morphological criteria (Figure 3A,B). To ensure the purity of recovered cells, immunocytochemistry (ICC) and immunofluorescence (IF) were performed to assess membrane expression of the pan-endothelial marker CD31 in GLA-ECs (Figure 3C) as well as in HD-ECs and HUVECs (Figure S4). The lymphatic endothelial phenotype of GLA-ECs was further characterized by D2-40 expression, assessed by ICC (Figure 3D), and by nuclear PROX1 expression detected by IF (Figure 3E–G). In addition, flow cytometric analysis of D2-40 expression was performed in both GLA-ECs and HD-ECs for comparison (Figure 3H and Figure S5, respectively). Flow cytometry documented that 98.2% of GLA-ECs were D2-40-positive compared with 63.6% of HD-ECs. Moreover, GLA-ECs displayed higher D2-40 expression levels, as indicated by mean fluorescence intensity (MFI) analysis.

### 3.4. NGS Assay

**Figure 4:**
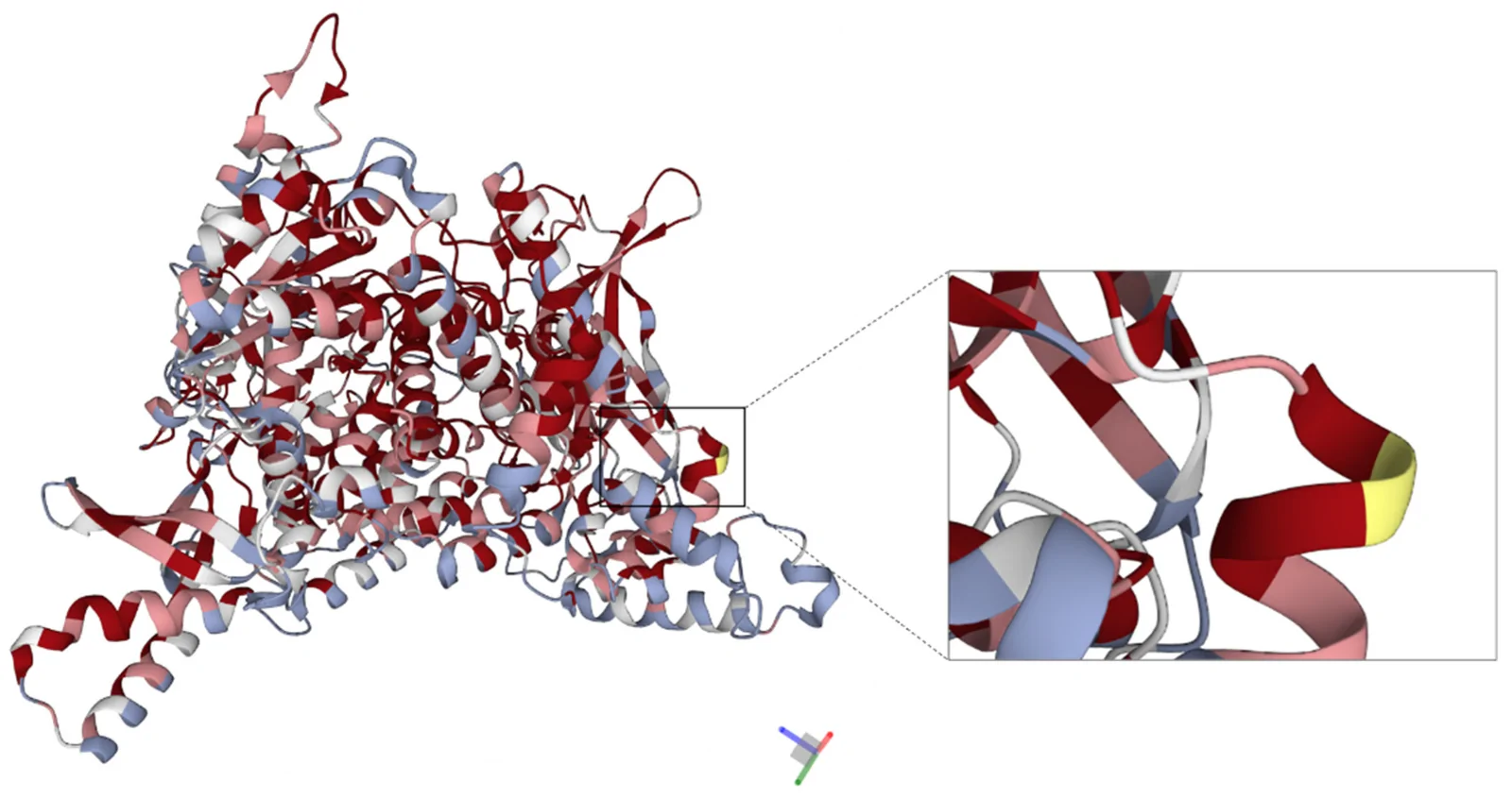
PI3Kca protein. Three-dimensional cartoon representation of PI3Kca showing Gln at position 546 (yellow) within the helical domain. The overall representative image of the protein was intentionally rotated to highlight the helical conformation. The right panel provides a magnified view of the region. Image adapted from the AlphaMissense database (https://alphamissense.hegelab.org/hotspot; accessed on 29 June 2026).

Through sequencing of the DNA extracted from isolated and expanded GLA-ECs, we detected a somatic missense variant in exon 10 of the *PIK3CA* gene with an allelic frequency of 12.08% (Table 3). In particular, we identified the previously reported *PIK3CA* c.1636G>A missense mutation, resulting in the p.Gln546Lys amino acid substitution within the helical domain of the p110α catalytic subunit of PI3K. This variant represents one of the most frequently reported *PIK3CA* mutations in generalized lymphatic anomaly (Figure 4) [9].

### 3.5. In Vitro Pharmacological Interference on GLA-EC Functional Properties

#### 3.5.1. Viability, Proliferation, and Apoptosis Analysis

**Figure 5:**
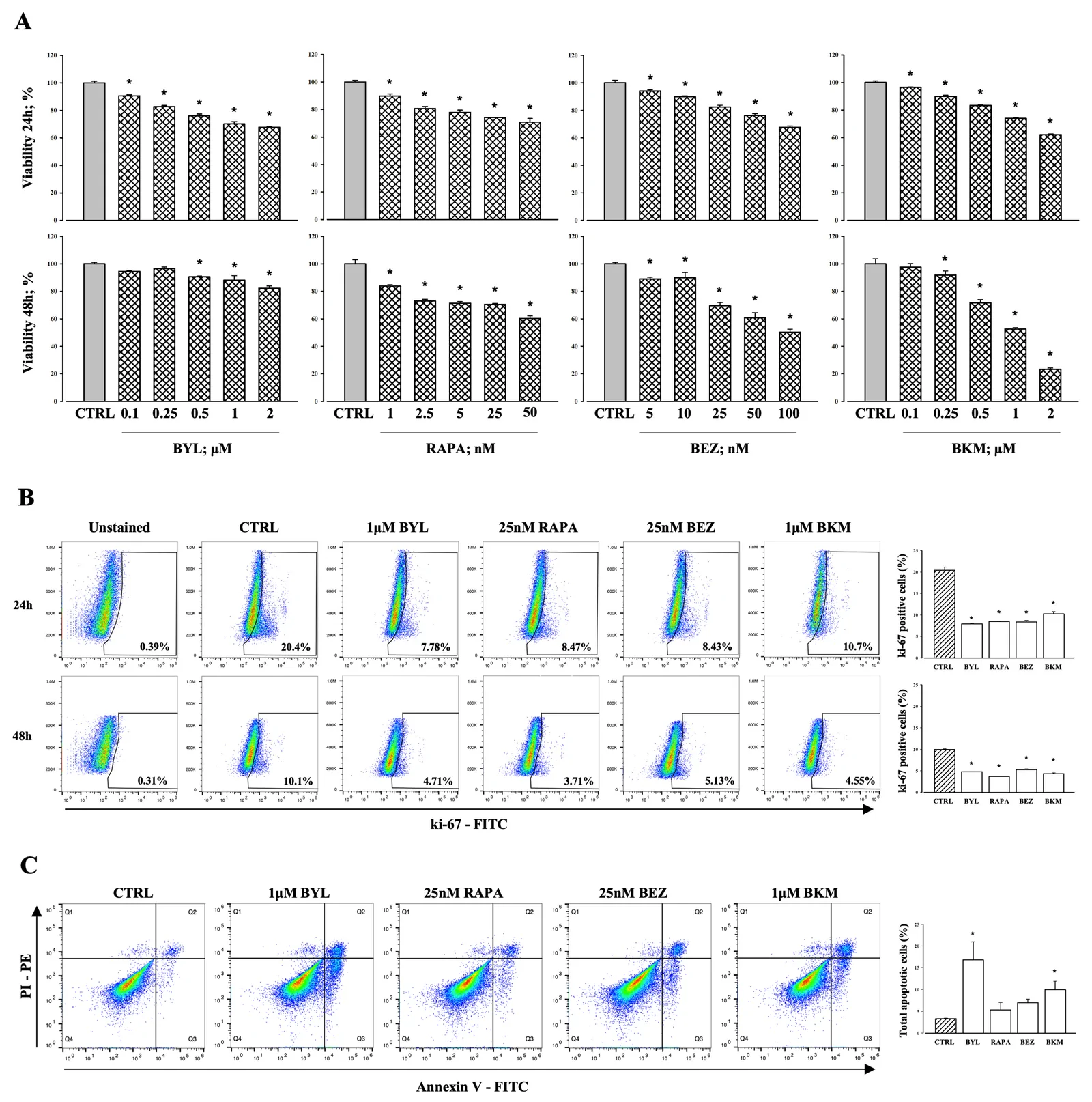
Effects of PI3K/AKT/mTOR pathway inhibitors on GLA-EC viability, proliferation, and apoptosis. (**A**) Representative histograms from MTT assays on GLA-ECs exposed for 24 and 48 h to increasing doses of alpelisib (BYL; PI3Kα inhibitor), rapamycin (RAPA; mTOR inhibitor), dactolisib (BEZ; dual PI3K/mTOR inhibitor), and buparlisib (BKM; pan-PI3K inhibitor). Pharmacological treatments affected GLA-EC viability in a dose- and time-dependent manner. Data are reported as mean ± standard error (SE) (*n* = 3); one-way ANOVA followed by Holm–Sidak’s *post hoc* test; * *p* < 0.05 vs. CTRL. (**B**) Representative flow cytometric analysis of ki-67 expression in GLA-ECs following treatment with 1 µM BYL, 25 nM RAPA, 25 nM BEZ, or 1 µM BKM for 24 and 48 h. The bar graph on the right shows the percentage of Ki-67-positive cells at each time point, reported as mean ± SE (*n* = 3); one-way ANOVA followed by Holm–Sidak’s *post hoc* test; * *p* < 0.05 vs. CTRL. (**C**) Representative Annexin V-FITC/propidium iodide (PI) flow cytometric analysis of GLA-ECs following 48 h of pharmacological treatment. The bar graph on the right shows total apoptosis, calculated as the sum of early (Q3; Annexin V^+^/PI^−^) and late (Q2; Annexin V^+^/PI^+^) apoptotic cell populations. Data are reported as mean ± SE (*n* = 3); one-way ANOVA followed by Holm–Sidak’s *post hoc* test; * *p* < 0.05 vs. CTRL. The mean percentages of cells within each quadrant (Q) are reported in Supplementary Table S2.

Based on the altered molecular background, GLA-ECs were exposed to BYL, RAPA, BEZ, or BKM, which target different components of the PI3K/AKT/mTOR pathway, and cell viability was tested by MTT after 24 and 48 h. As shown in Figure 5A, all four inhibitors significantly reduced GLA-EC viability in a dose- and time-dependent manner compared with untreated controls (*p* < 0.05), although the effect of BYL was less pronounced after 48 h.

To further investigate the effects of PI3K/AKT/mTOR pathway inhibition on GLA-EC proliferation, ki-67 expression was evaluated by flow cytometry following treatment with 1 µM BYL, 25 nM RAPA, 25 nM BEZ, or 1µM BKM for 24 and 48 h. Quantitative analysis of three independent experiments showed that all four inhibitors reduced the percentage of Ki-67-positive GLA-ECs compared with the corresponding untreated controls at both time points, supporting an inhibitory effect on cell proliferation (Figure 5B). At 24 h, ki-67-positive cells decreased from 20.4% in untreated GLA-ECs to 7.78%, 8.47%, 8.43%, and 10.7% following BYL, RAPA, BEZ, and BKM treatment, respectively. At 48 h, ki-67-positive cells accounted for 10.1% of untreated GLA-ECs and 4.71%, 3.71%, 5.13%, and 4.55% of BYL-, RAPA-, BEZ-, and BKM-treated cells, respectively.

Finally, to determine whether the reduction in cell viability was associated with apoptotic cell death, Annexin V/PI flow cytometric analysis was performed after 48 h of pharmacological treatment. Quantitative analysis of total apoptosis, defined as the sum of early (Annexin V^+^/PI^−^) and late (Annexin V^+^/PI^+^) apoptotic cells, was performed across three independent experiments. Total apoptosis increased following BYL and BKM treatment, with a more pronounced effect observed after BYL exposure, whereas RAPA and BEZ induced only minor changes compared with untreated GLA-ECs (Figure 5C). The mean percentages of cells within each quadrant of the Annexin V/PI analysis and the corresponding total apoptosis rates are reported in Supplementary Table S2.

#### 3.5.2. Migration Assay

**Figure 6:**
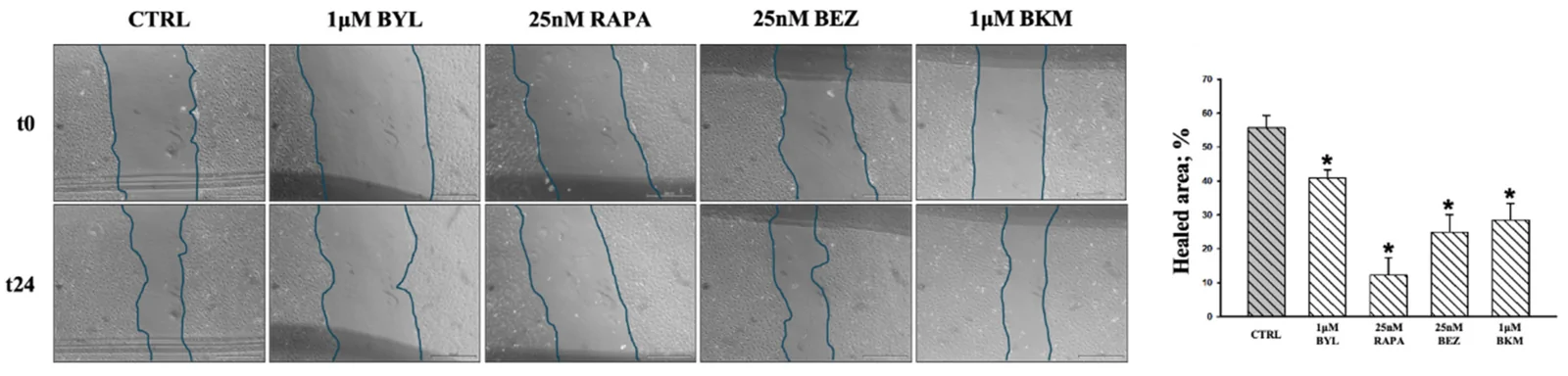
Wound-healing assay. Representative images of GLA-ECs captured during the wound-healing assay at 0 and 24 h following pharmacological treatments. Dark marks of the felt-tip pen were used to monitor the same area over time. Scale bars = 500 μm. Percent migration with respect to untreated cells (CTRL) is reported in the bar graph as mean ± SE (*n* = 3); one-way ANOVA followed by Holm–Sidak’s *post hoc* test; * *p* < 0.05 vs. CTRL.

To evaluate the effects of PI3K/AKT/mTOR pathway inhibition on GLA-EC migration, cells were treated with 1 µM BYL, 25 nM RAPA, 25 nM BEZ, or 1 µM BKM and assessed using a scratch wound-healing assay. Twenty-four hours after the scratch, GLA-ECs in control medium closed 55.6% of the wound area, compared with 40.9%, 12.2%, 24.8%, and 28.0% following BYL, RAPA, BEZ, or BKM exposure, respectively (Figure 6).

**Figure 7:**
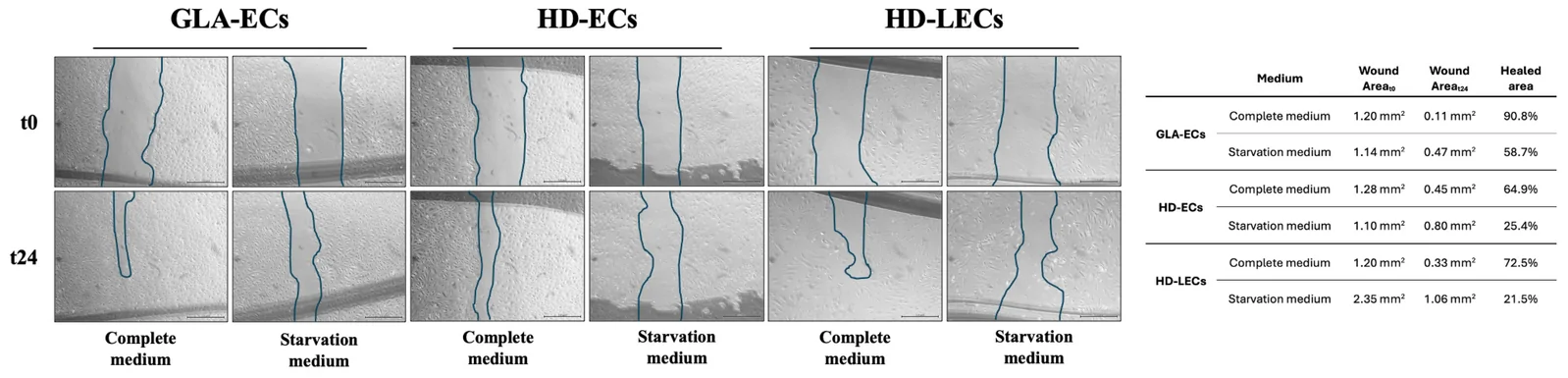
Impact of starvation on endothelial cell migration. GLA-ECs, HD-ECs, and HD-LECs were cultured either in complete or starvation medium to assess cell migration and wound closure ability under different nutritional conditions. Dark marks of the felt-tip pen were used to monitor the same area over time. Scale bars = 500 μm. The table on the right reports the wound area at 0 and 24 h and the corresponding percentage of healed area for each cell population under complete and starvation conditions.

Additionally, the migratory capacity of GLA-ECs, HD-ECs, and HD-LECs was evaluated at 0 and 24 h in either complete or starvation medium. Notably, GLA-EC monolayers retained substantial migratory capacity even under starvation conditions (Figure 7). In contrast, both HD-EC and HD-LEC monolayers exposed to starvation medium virtually lost their migratory capacity and were unable to efficiently close the wound, as demonstrated by comparison of the wound areas at 0 and 24 h (Figure 7).

#### 3.5.3. Lymphangiogenic Assay

**Figure 8:**
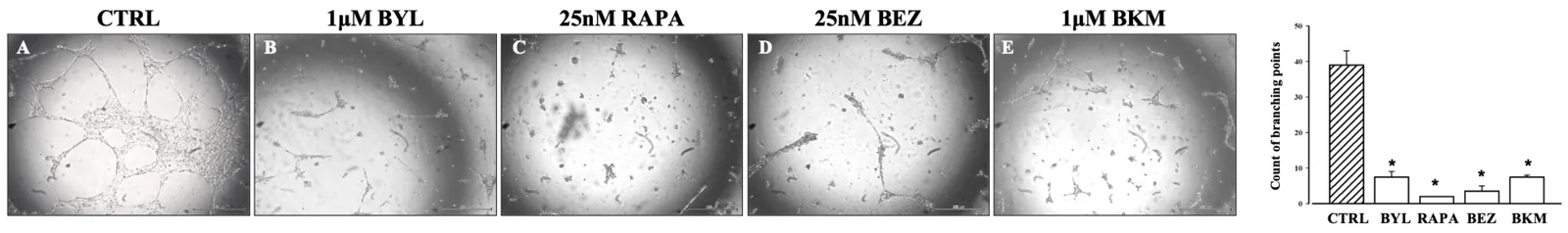
Matrigel assay. Representative images of the tube formation ability. GLA-ECs (2 × 10^4^ cells/100 μL per well) were gently layered on top of Matrigel^®^ in complete medium alone (CTRL; (**A**)), or with 1 µM BYL (**B**), 25 nM RAPA (**C**), 25 nM BEZ (**D**), or 1 µM BKM (**E**). Images were captured after 24 h. Scale bars = 500 μm. The number of branching nodes is reported in the bar graph as mean ± SE (*n* = 3); one-way ANOVA followed by Holm–Sidak’s *post hoc* test; * *p* < 0.05 vs. CTRL.

Analysis of capillary-like structure formation on Matrigel^®^ confirmed and extended the results obtained with the wound-healing assay. Specifically, all four PI3K/AKT/mTOR pathway inhibitors markedly impaired the lymphangiogenic capacity of GLA-ECs. While GLA-ECs cultured in complete medium formed a well-organized capillary-like network, pharmacological treatment resulted in incomplete and truncated tubular structures, accompanied by cell clustering (Figure 8). Quantitative analysis confirmed a significant reduction in the number of branching points following treatment with all four inhibitors compared with untreated GLA-ECs (Figure 8).

**Figure 9:**
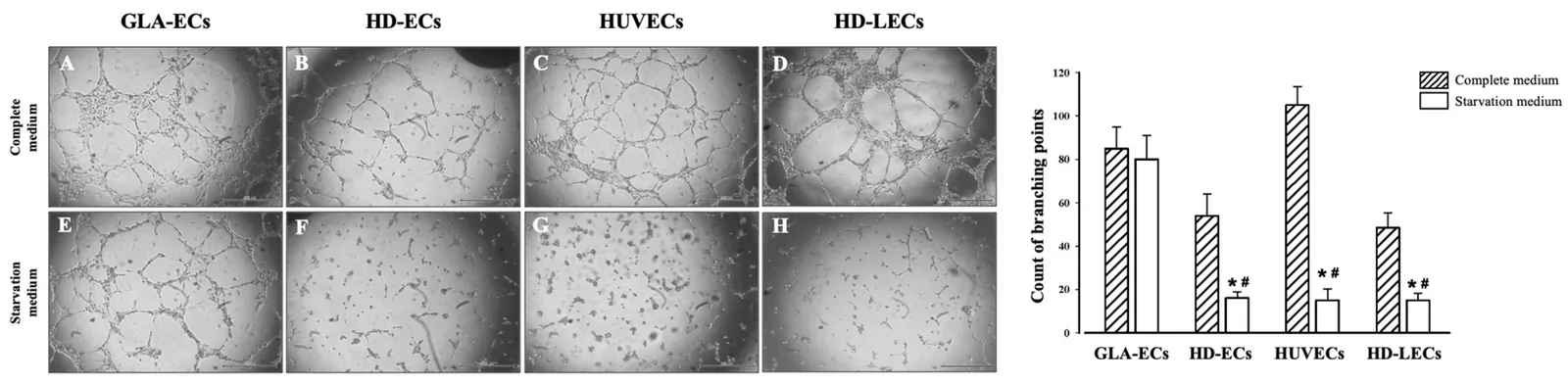
Lymphangiogenic capacity under starvation conditions. To assess mutation-dependent lymphangiogenic capacity, GLA-ECs, HD-ECs, HUVECs, and HD-LECs were serum-starved for 18 h and subsequently seeded on Matrigel^®^ in either complete medium (**A**–**D**) or starvation medium (**E**–**H**) (see Section 2.11). Images were acquired after 6 h to assess tube-forming ability. Scale bars = 500 μm. The number of branching nodes is reported in the bar graph as mean ± SE (*n* = 3). Statistical analysis was performed using two-way ANOVA followed by Holm–Sidak’s multiple comparisons test; * *p* < 0.05 vs. the corresponding complete-medium condition; # *p* < 0.05 vs. GLA-ECs under starvation conditions.

In addition, to investigate the ability of GLA-ECs to maintain lymphangiogenic activity under growth factor deprivation, GLA-ECs, HD-ECs, HUVECs, and HD-LECs were starved for 18 h, plated on Matrigel^®^ in either complete or starvation medium, and evaluated after 6 h. Notably, all four endothelial cell populations formed an extensive tubular-capillary network under complete culture conditions. Conversely, under starvation conditions, only GLA-ECs retained the ability to form a well-organized network characterized by branching points and complete tubular structures, whereas HD-ECs, HUVECs, and HD-LECs failed to maintain organized network formation (Figure 9).

#### 3.5.4. Immunoblotting Analysis

**Figure 10:**
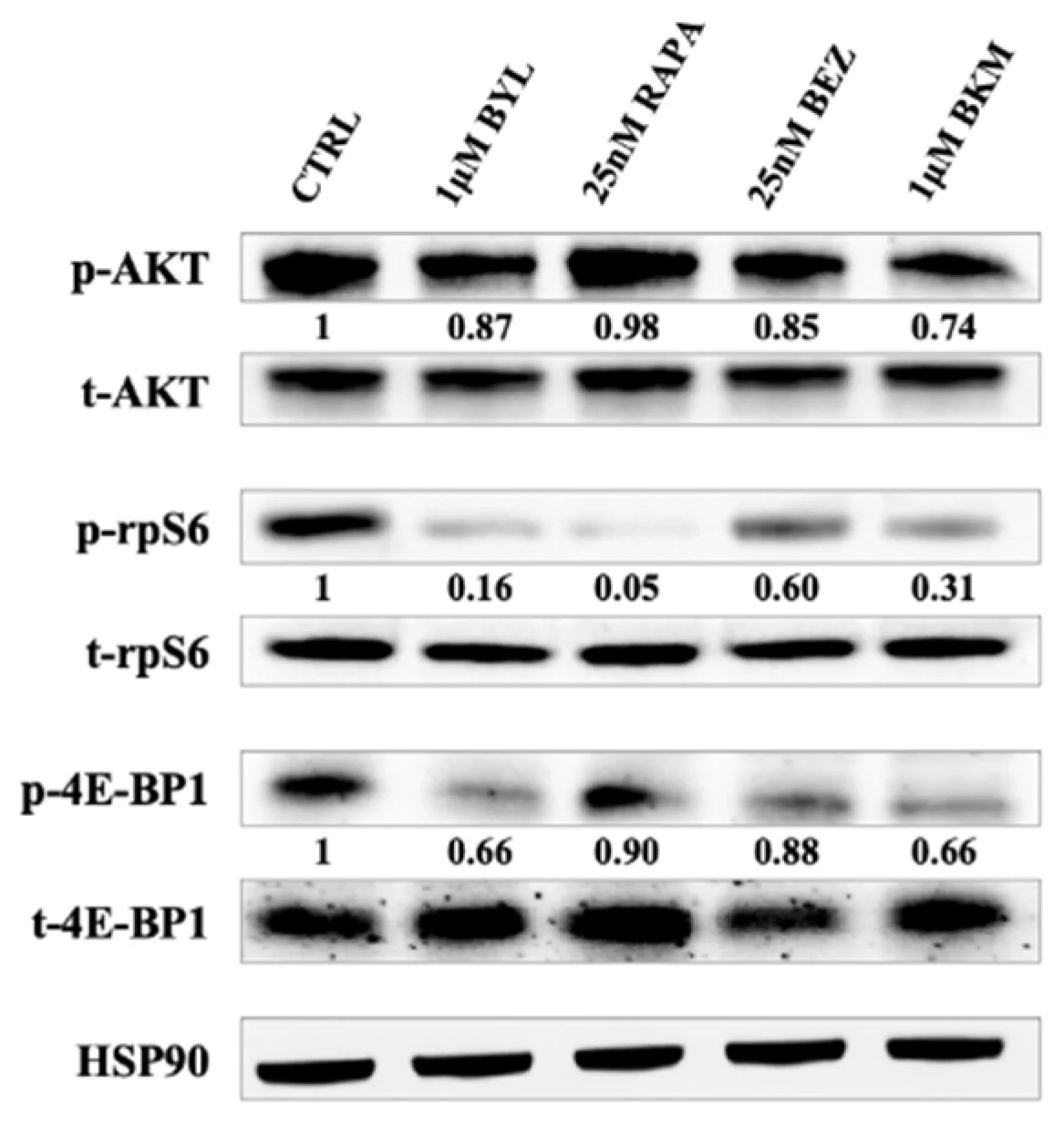
Immunoblotting analysis. Representative blots of p-AKTSer473, total AKT, p-rp-S6Ser235/236, total rp-S6, p-4E-BP1Ser65, and total 4E-BP1 in GLA-EC cells following 90 min treatment with 1 μM BYL, 25 nM RAPA, 25 nM BEZ, or 1 μM BKM. HSP90 was used as a loading control. Densitometric values for phosphorylated proteins normalized vs. control (HSP90) are reported at the bottom of each blot.

In line with the data obtained from histological, genetic, and functional analysis, we sought to demonstrate and corroborate the effects of PI3K/AKT/mTOR inhibition on GLA-ECs from a mechanistic point of view. Western blot assay demonstrated decreased phosphorylation of ribosomal protein S6 at Ser235/236 and 4E-BP1 at Ser65, two downstream key substrates of the PI3K/AKT/mTOR axis, following all pharmacological treatments (Figure 10). Additionally, treatment with BYL, BEZ, or BKM effectively reduced phosphorylation levels of p-AKT at Ser473. Conversely, selective mTORC1 inhibition by rapamycin was unable to blunt AKT at Ser473, likely reflecting the unleashed activation of the mTORC2 complex.

To extend the analysis to normal lymphatic endothelial cells, the effects of the same pharmacological treatments were evaluated in HD-LECs. Western blot analysis showed a reduction in p-AKT^Ser473^ levels following all treatments, with a more pronounced effect for BEZ and BKM. Likewise, p-rpS6^Ser235/236^ levels were also reduced by all treatments, with the strongest inhibition observed following RAPA exposure (Figure S6).

## 4. Discussion

In recent years, advances in genetic approaches have enabled the identification of single somatic mutations affecting key regulators of intracellular signaling pathways involved in endothelial cell proliferation, survival, migration, and angiogenesis. These findings have significantly improved the understanding of the pathogenesis of several vascular anomalies and have supported the development of targeted therapeutic strategies [5,7,8]. In particular, the *PIK3CA* gene mutations, which are commonly observed in several neoplastic conditions alongside other driver alterations, have also been frequently identified in isolated or multifocal lymphatic malformations. These mutations result in constitutive hyperactivation of the PI3K/AKT/mTOR signaling pathway [31,32].

Here, we report an extensive histological and immunohistochemical characterization of the generalized lymphatic anomaly at the tissue level. Our data document that lymphatic endothelial cells express characteristic endothelial and lymphatic-specific markers, such as CD31, podoplanin/D2-40, LYVE-1, and PROX1, thereby confirming the lymphatic identity of the vascular lesion. Moreover, in line with previous reports, the absence of co-expression of WT1 and nestin in lymphatic endothelial cells supports the non-neoplastic nature of the vascular proliferation [28,29,30]. The abundant, dysmorphic, and dilated vessels observed in GLA may reflect a low-grade endothelial cell proliferation, as supported by nuclear ki-67 labeling. These findings were further supported by the immunohistochemical analysis of the phosphorylation status of the PI3K/AKT/mTOR pathway at the tissue level. Using this approach, we demonstrated activation of key downstream effectors of the PI3K/AKT/mTOR pathway, which regulates growth, proliferation, survival, migration, and angiogenesis. Importantly, double immunofluorescence demonstrated co-localization of D2-40 with phospho-mTOR and phospho-rpS6, confirming that pathway activation occurs within the lymphatic endothelial compartment. The pathogenesis of GLA is driven by recurrent hotspot missense mutations in *PIK3CA*, typically affecting the helical or kinase domains of the protein. Through NGS analysis, we identified the c.1636C>A variant, resulting in the p.Gln546Lys substitution, which has been reported to be more frequently associated with GLA compared to other lymphatic malformations [7,9].

We next compared GLA-ECs with normal endothelial controls, including HD-ECs, HUVECs, and commercially available HD-LECs, to investigate whether the constitutive activation of the PI3K/AKT/mTOR pathway was associated with an altered functional phenotype. Notably, GLA-ECs retained both migratory and capillary-like network-forming capacity under starvation conditions, whereas the normal endothelial populations, including HD-LECs, showed a marked impairment of these functions. The persistence of these functional properties in GLA-ECs, but not in normal endothelial cells, suggests an enhanced ability of GLA-derived cells to maintain functional activity under growth factor-deprived conditions, consistent with a gain-of-function phenotype associated with constitutive PI3K/AKT/mTOR pathway dysregulation and reduced dependence on exogenous growth stimuli. The molecular mechanisms underlying this response to growth factor deprivation remain to be elucidated. In particular, the potential contribution of adaptive stress response mechanisms, including autophagy, represents an important unanswered question that warrants dedicated investigation.

The GLA-EC line was subsequently subjected to in vitro functional assays following pharmacological treatment with four drugs already approved for clinical use or currently under clinical investigation, namely, rapamycin, alpelisib, buparlisip, and dactolisib. In particular, rapamycin, initially introduced as an immunosuppressive agent for organ transplantation and later approved for the treatment of tuberous sclerosis complex disease, bone sarcoma, and lymphoma, has also shown promising clinical activity in patients with lymphatic lesions [21,33,34]. Alpelisib was approved by the US Food and Drug Administration (FDA) in May 2019 in combination with fulvestrant for *PIK3CA*-mutated breast cancer and has subsequently been used alone as a well-tolerated and effective treatment for PROS [35,36,37,38,39,40]. Buparlisib, a class I PI3K pan-inhibitor, has been employed in combination with paclitaxel for squamous cell carcinoma of the head and neck and with fulvestrant for hormone receptor-positive breast cancer harboring *PIK3CA* mutations [37,41,42,43]. Finally, dactolisib, a dual inhibitor of PI3K and mTOR kinase, was originally developed for cancer treatment and designed to trigger tumor cell apoptosis. However, it is currently under investigation in the experimental and preclinical phase due to toxicity concerns [44,45]. All PI3K/AKT/mTOR inhibitors tested reduced GLA-EC viability in a dose- and time-dependent manner, with effects observed at both 24 and 48 h. Flow cytometric analysis of ki-67 demonstrated that all four inhibitors reduced the proliferative fraction of GLA-ECs at both time points, supporting an antiproliferative effect of PI3K/AKT/mTOR pathway inhibition. Annexin V/PI analysis further showed that the reduction in cell viability was accompanied by increased apoptosis following BYL and BKM treatment, whereas RAPA and BEZ produced only minor changes in apoptotic cell populations under the experimental conditions used. These findings suggest that the reduction in GLA-EC viability may result from distinct combinations of antiproliferative and pro-apoptotic effects depending on the pharmacological target. In parallel, all four inhibitors significantly impaired GLA-EC migratory and lymphangiogenic capacities, further supporting the functional relevance of PI3K/AKT/mTOR signaling in sustaining the pathological endothelial phenotype. Together, these findings indicate that pharmacological inhibition of this pathway affects multiple GLA-EC functions, including viability, proliferation, migration, and capillary-like network formation. These cellular properties are critical drivers of lymphatic lesion size and survival and, when left unchecked, likely contribute to the progressive enlargement of the lesions observed during patient growth.

From a mechanistic point of view, treatment with all four drugs resulted in decreased phosphorylation of 4E-BP1 at Ser65, a 5′cap-dependent translation initiation factor, together with an even more pronounced reduction in the phosphorylation of ribosomal protein S6 (Ser235/236), a downstream effector of the S6K axis involved in protein synthesis, cell growth, and proliferation. In addition, a marked reduction in AKT phosphorylation at Ser473 was observed following treatment with PI3K pathway inhibitors, consistent with effective attenuation of upstream signaling. This effect was particularly evident following treatment with the dual PI3K/mTOR inhibitor dactolisib, indicating direct targeting of mTORC2 activity. In contrast, acute rapamycin treatment efficiently suppressed mTORC1 downstream effectors 4E-BP1 and rp-S6 but did not affect AKT phosphorylation at Ser473. These findings are consistent with the selective inhibition of mTORC1 by rapamycin and indicate that mTORC2-dependent AKT activation remains largely preserved under acute treatment conditions. Notably, pharmacological modulation of the pathway was also observed in normal HD-LECs, indicating that the effects of these inhibitors are mutation-independent and not restricted to *PIK3CA*-mutated cells. This finding is consistent with their mechanism of action, as these compounds directly interfere with components of the PI3K/AKT/mTOR signaling pathway that are also physiologically active in normal lymphatic endothelial cells. Nevertheless, in GLA-ECs, pathway inhibition occurs in the context of constitutive signaling dysregulation associated with the identified *PIK3CA* mutation. The observed effects on normal HD-LECs, therefore, raise an important issue regarding therapeutic selectivity and the potential impact of sustained pathway inhibition on normal lymphatic endothelium. Although these molecular effects do not reflect cellular toxicity, they highlight the importance of defining an appropriate therapeutic window for future treatments, capable of effectively controlling aberrant signaling in mutant cells while minimizing unwanted effects on normal tissues [21,38].

### Study Limitations

Our goal was to contribute to the growing understanding of the molecular basis underlying the spectrum of vascular anomalies. Although the use of patient-derived primary GLA-ECs represents a strength of the present study, the availability of cells from a single patient limits the generalizability of our findings and warrants validation in additional GLA cases. Further in vivo and clinical studies will be necessary to confirm the translational relevance of our observations and to evaluate long-term efficacy, safety, and potential resistance mechanisms associated with PI3K/AKT/mTOR-targeted therapies [38,46].

## 5. Conclusions

Precision diagnostics in pathology, integrated with next-generation sequencing, can effectively improve the classification of vascular anomalies. Cell culture models remain a cornerstone for evaluating the functional and mechanistic effects of novel therapeutic compounds. Multidimensional approaches may facilitate the implementation of personalized targeted therapies, particularly for patients with recurrent disease or when surgical treatment is challenging or not feasible [47].

## Figures and Tables

**Table 1:** Primary antibodies used for the IHC assay.

Marker	Source	Company	Antigen Retrieval	Processing System Tool	Dilution	Time (min)	Temp. (°C)	Clone	Ref. cod.
CD31	Ms	Ventana-Roche	15′ MW	*	ready to use	28	37	JC70	05463475001
Podoplanin/D2-40	Ms	Cell Marque	15′ MW	*	ready to use	28	37	D2-40	322M-18
ki-67	Ms	Agilent	15′ MW	*	1:100	28	37	Mib-1	IR626
LYVE-1	Rb	Abcam	35′ bath 95 °C	**	1:100	o/n	4	polyclonal	ab36993
VEGFR-2	Ms	SCBT	35′ bath 95 °C	**	1:60	60	RT	A-3	sc-6251
VEGFR-3	Ms	Abcam	35′ bath 95 °C	**	1:100	o/n	4	polyclonal	ab14828
PROX1	Rb	Origene	35′ bath 95 °C	**	1:500	o/n	4	polyclonal	DP3516P
Nestin	Rb	Millipore	15′ MW	**	1:1000	45	RT	polyclonal	AB5922
WT1	Ms	Ventana-Roche	15′ MW	*	ready to use	28	37	6F-H2	05435706001
p-mTOR^Ser2448^	Rb	CST	15′ MW	**	(IF) 1:50	o/n	4	D9C2	#5536
p-mTOR^Ser2448^	Rb	CST	35′ bath 95 °C	**	(IHC) 1:250	o/n	4	49F9	#2976
p-rpS6^Ser235/236^	Rb	CST	35′ bath 95 °C	**	1:400	o/n	4	D57.2.2	#4858
p-AKT^Ser473^	Rb	CST	35′ bath 95 °C	**	1:150	o/n	4	736E11	#3787
p-p70S6K^Thr389^	Rb	CST	35′ bath 95 °C	**	1:50	o/n	4	108D2	#9234
p-4E-BP1^Ser65^	Rb	CST	35′ bath 95 °C	**	1:100	o/n	4	74A9	#9456

* BenchMark Ultra (Ventana–Roche, Tucson, AZ, USA); ** manual; MW = microwave oven; Ms = mouse; Rb = rabbit; RT = room temperature; o/n = overnight; CST = Cell Signaling Technology; SCBT = Santa Cruz Biotechnology.

**Table 2:** Pharmacological inhibitors.

Compound	Alternative Name	Target	Abbreviation Used
Alpelisib	BYL719 (NVP-BYL719)	Selective PI3Kca inhibitor	BYL
Buparlisib	BKM120 (NVP-BKM120)	Class I pan-PI3K inhibitor	BKM
Rapamycin	Sirolimus	mTORC1 inhibitor	RAPA
Dactolisib	BEZ235 (NVP-BEZ235)	Dual PI3K/mTOR inhibitor (mTORC1/mTORC2)	BEZ

**Table 3:** Genetic characterization of the GLA-EC line.

Sex	Age (Month)	Site	Gene	Exon	cDNA	Aa	rsID	Allelic Freq	Clin Var
F	2.5	thoracic wall	PIK3CA	10	c.1636C>A	p.Gln546Lys	rs121913286	12.08%	Pathogenic

## Data Availability

The original contributions presented in this study are included in the article/Supplementary Material. Further inquiries can be directed to the corresponding authors.

## Supplementary Materials

The following supporting information can be downloaded at https://www.mdpi.com/article/10.3390/cells15201860/s1, Figure S1: Comparative immunofluorescence analysis; Figure S2: Immunofluorescence analysis of PI3K/AKT/mTOR pathway activation in lymphatic endothelial cells of GLA tissue; Figure S3: Immunohistochemical analysis of foreskin tissue; Figure S4: Morphologic and phenotypic characterization of endothelial cell lines; Figure S5: Flow cytometric characterization of D2-40 expression in HD-ECs; Figure S6: Immunoblotting analysis; Table S1: Genes included in the NGS panel; Table S2: Distribution of viable, apoptotic, and necrotic GLA-ECs following pharmacological treatment.
