# Supplementary material for: Targeting PI3K/AKT/mTOR Pathway Exerts Inhibitory Effects on Endothelial Cells from Generalized Lymphatic Anomaly
Source: Cells. 2026 Oct 13;15(20):1860. doi: 10.3390/cells15201860 (PMC13703100; doi:10.3390/cells15201860)
Supplement: Supplementary file 1 [file cells-15-01860-s001.zip › Supplementary Figures Legend.pdf]

Figure S1. Comparative immunofluorescence analysis. Representative immunofluorescence images of p-mTOR^{Ser2448} expression in human generalized lymphatic anomaly (GLA) tissue and foreskin tissue used as negative control. For each sample, bright field, DAPI nuclear staining (blue), p-mTOR^{Ser2448} (green) and merged channels are shown. p-mTOR^{Ser2448} immunoreactivity is detected in GLA tissue, whereas foreskin tissue displays negligible fluorescence signal, confirming the specificity of the staining. Scale bar= 100 µm.

Figure S2. Immunofluorescence analysis of PI3K/AKT/mTOR pathway activation in lymphatic endothelial cells of GLA tissue. Representative double immunofluorescence images illustrating D2-40-positive lymphatic endothelial cells (red) labelled by phospho-mTOR^{Ser244}8 (green, upper panels) and phospho-rpS6$^{Ser235/236}$ (green, lower panels). Nuclei were counterstained with DAPI (blue). Merged images show co-localization (yellowish) of D2-40 with phospho-mTOR^{Ser2448} and phospho-rpS6$^{Ser235/236}$, respectively. Scale bars= 60 µm.

Figure S3. Immunohistochemical analysis of foreskin tissue. Representative images of immunohistochemical staining of foreskin tissue showing lack of phosphorylated 4E-BP1^{Ser65} (A), p70S6K^{Thr389} (B), and AKT^{Ser473} (C) signals on vascular endothelial cells. Scale bar= 100 µm.

Figure S4. Morphologic and phenotypic characterization of endothelial cell lines. The purity of primary cultures of human umbilical vein endothelial cells (HUVECs) and human normal dermal-derived endothelial cells from foreskin (HD-ECs) was determined according to morphologic criteria (A and C) and ensured by the immunofluorescence detection of the pan-endothelial marker CD31 (green; B and D). Nuclei are counterstained by DAPI (blue). Scale bar: A and C= 500 µm; B and D= 50 µm.

Figure S5. Flow cytometric characterization of D2-40 expression in HD-ECs**.** Flow cytometric analysis showed that 63.6% of HD-ECs were D2-40-positive, with a median fluorescence intensity (MFI) of 7145. Therefore, HD-ECs are consistent with a mixed blood and lymphatic endothelial cell population. The unstained control and D2-40-stained population are shown in gray and green, respectively.

Figure S6. Immunoblotting analysis. Representative blots of p-AKT^{Ser473}, total AKT, p-rp-S6$^{Ser235/236}$, and total rp-S6 in HD-LECs following 90-minutes treatment with 1µM BYL, 25nM RAPA, 25nM BEZ, or 1µM BKM. HSP90 was used as loading control. Densitometric values normalized vs. control (HSP90) are reported at the bottom of each blot.
