# Supplementary material for: Targeting PI3K/AKT/mTOR Pathway Exerts Inhibitory Effects on Endothelial Cells from Generalized Lymphatic Anomaly
Source: Cells. 2026 Oct 13;15(20):1860. doi: 10.3390/cells15201860 (PMC13703100; doi:10.3390/cells15201860)
Supplement: Supplementary file 1 [file cells-15-01860-s001.zip › Supplementary Tables.pdf]

Table S1. Genes included in the NGS panel

AKT1	EGFR	FGFR4	KRAS	NTRK2	RET
ALK	ERBB2	GNA11	MAP2K1	NTRK3	ROS1
AR	ERBB3	GNAQ	MET	PDGFRA	SMAD4
BRAF	ERBB4	GNAS	MTOR	PIK3CA	SMO
CDK4	ESR1	HRAS	NF1	POLE	SKT11
CDKN2A	FGFR1	IDH1	NRAS	PTEN	TERT promoter
CTNNB1	FGFR2	KEAP1	NRG1	RAF1	TP53
DDR2	FGFR3	KIT	NTRK1	RB1	TSC1

Table S2. Distribution of viable, apoptotic, and necrotic GLA-ECs following pharmacological treatment

	**Necrotic Q1 (%)**	**Late apoptosis Q2 (%)**	**Early apoptosis Q3 (%)**	**Viable Q4 (%)**	**Total apoptosis Q2+Q3 (%)**
**CTRL**	0.72±0.09	2.21±0.24	1.09±0.15	96.00±0.17	3.30±0.09
**BYL**	0.71±0.11	7.26±1.65	9.52±0.94	82.50±2.35	16.78±2.42
**RAPA**	0.45±0.04	2.79±0.53	2.54±0.45	94.23±0.93	5.33±0.96
**BEZ**	0.44±0.09	3.32±0.79	3.68±0.56	92.57±0.56	7.00±0.48
**BKM**	1.05±0.08	4.57±0.72	5.42±1.76	88.97±1.19	9.98±1.11
